# Management of penetrating cardiac injuries in the Department of surgery, Mohamed Thahar Maamouri Hospital, Tunisia: report of 19 cases

**Published:** 2012-03-22

**Authors:** Sonia Baccari Ezzine, Mahdi Bouassida, Mechaal Benali, Mosaab Ghannouchi, Fethi Chebbi, Sélim Sassi, Mohamed Mongi Mighri, Hassen Touinsi, Sadok Sassi

**Affiliations:** 1 Department of surgery, Mohamed Thahar Maamouri Hospital, Nabeul, Tunisia; 2 Department of reanimation, Mohamed Thahar Maamouri Hospital, Nabeul, Tunisia

**Keywords:** Cardiac injury, stab wounds, tamponnade, hemorrhage, cardiac repair, Tunisia

## Abstract

The goal of this paper is to discuss how to ameliorate the management of penetrating cardiac injuries in general surgery department. An algorithm for the initial assessment of penetrating injuries in cardiac box, based on our own experience, is presented. This was a retrospective study of 19 patients undergoing thoracotomy for penetrating cardiac injuries, managed in the department of general surgery of Nabeul-Tunisia, between 1994 and 2010. The mean age of patients was 25 years old. Sex ratio was 8,5. All patients had cardiac injury resulting from stab wounds inside of the pericardium. 42% of them were critically unstable, 21% had cardiac tamponnade. All these patients were quickly transferred to the operating room without any other investigations. 37% of patients were hemodynamically stable and underwent additional investigations. The management of penetrating cardiac injuries is possible in a general surgery department, but it requires a rapid prehospital transfer, a yet thorough physical examination along with efficient surgical management, all done in minimal time.

## Introduction

Despite the improvement in trauma care, penetrating injuries to the heart continue to be a source of significant mortality. Most penetrating cardiac injuries are secondary to acts of violence. Penetrating wounds from sharp objects is associated in general with a better outcome than those resulting from gunshot. All Tunisian series noted that penetrating stab wounds are more frequent as a result of the fact that the possession of fire arms is illegal in Tunisia. A rapid diagnosis and surgical intervention can salvage patients who would otherwise be lost. All general surgeons should be capable of recognizing these injuries and intervening if a cardiothoracic surgeon is not immediately available. The goal of our study was to analyze the therapeutic pitfalls posed by penetrating cardiac injuries, based on our own experience and compared with the recent literature on the subject.

## Methods

Our retrospective series consisted of 19 consecutive patients undergoing thoracotomy for penetrating cardiac injuries, managed in the department of general surgery of Nabeul during the seventeen-year period between the 1st of January 1994 and the 31st of December 2010. Among them, 17 patients had penetrating injury of the heart and two patients had pericardial injury and hematoma. Patients with thoracotomy performed for blunt trauma and cases with iatrogenic lacerations of the heart were not included. The decision to perform thoracotomy was based on the discretion of the attending general surgeon.

Data collected included patient age and sex, mechanism of injury (stab wound in all cases), method of hospital transportation (fire rescue, police and private vehicles), initial clinical presentation and hemodynamic findings, preoperative investigations (chest radiographs, electrocardiogram, echocardiography, CT scan) if present, surgical modalities for cardiac repair, length of hospital stay, hospital survival, and cardiac outcome.

## Results

Over a seventeen-year period (1994–2010), nineteen victims of trauma sustaining penetrating cardiac injury arrived alive at the department of general surgery of Nabeul. Patients were primarily young (the mean age was 25). There were 17 men and 2 women. 65% of the patients were transported by police, 11% by emergency medical system, and 24% by private vehicle. 11% of study patients underwent prehospital endotracheal intubation.

First clinical evaluation was done in the emergency room and showed: All patients had cardiac injury resulting from stab wounds inside of the pericardium (18 patients in the left hemi thorax and only one patient in the right hemi thorax); 42% of patients were critically unstable (they had systolic blood pressure ≤90 mm hg), 10% of them were unconscious. 21% of patients had cardiac tamponade with Beck's triad. These patients underwent endotracheal intubation, venous lines were obtained, chest radiographs were done in emergency room and patients were quickly transferred to the operating room without any other investigations; 37% of patients were hemodynamically stable and underwent additional investigations.

**Figure 1:**
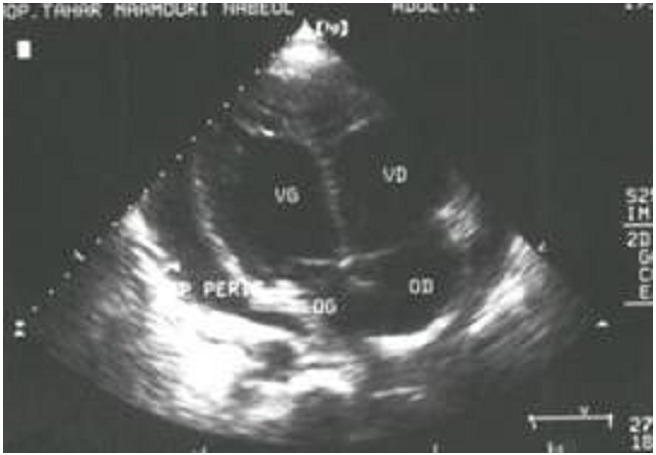
Echocardiography practiced in emergency confirming the hemopericardium

Chest radiographs were done to all our patients; it showed a haemopneumothorax in 35% of cases, a haemothorax in 23% of cases, an enlargement of the cardiac silhouette in 23% of cases. Chest radiographs were normal in the other cases. Electrocardiogram was done to 4 patients; it showed a pulseless electrical activity in one case and a sinus tachycardia in three cases. Echocardiography detected hemopericardium in 5 cases and was normal in one case (Figure 1).

**Figure 2:**
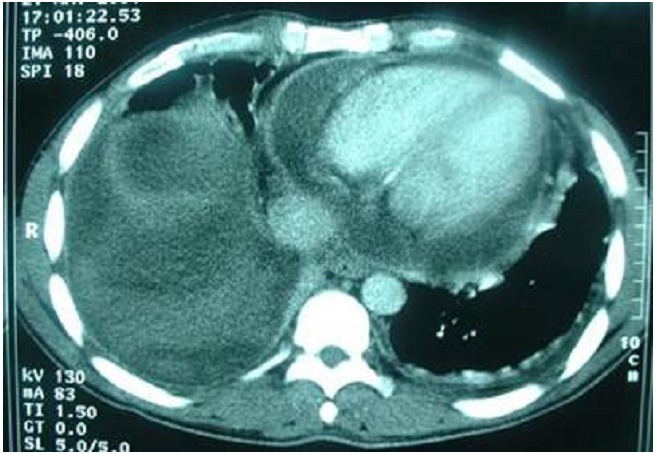
CT scan showed a hemopericardum associated with an important haemothorax

In the two patients who had a CT scan, a hemopericardium was detected (Figure 2). 18 patients were operated in emergency condition the same day of the traumatism (intermediate duration between the occurence of the wound and the intervention was of 45 minutes). Only one patient having a stable hemodynamic state and a normal cardiac echography, was hospitalized and supervised. The hemodynamic deterioration of the state on the 7th day required the practice of a thoracic scanner which objectified the existence of pericardial effusion therefore the patient was operated. The left antero-lateral thoracotomy was the way of choice for our team.

In concern of operative findings, the left ventricle was injured in 2 cases, the right ventricle in 14 cases, and the right auricle in one case. There was a contusion without myocardial lesion in 2 cases In addition to cardiac wounds; there were wounds of the pulmonary parenchyma in 3 cases, a section of the internal thoracic artery in one case, a section of the intercostal pedicle in 2 cases and diaphragmatic injury in 2 cases. The pericardium was opened in all the cases. Then, a temporary digital homeostasis was performed. The suture of the cardiac wounds by separate points, reinforced by the pericardium, was done with non-resorbable 3/0 wire. Finally, pericardial stitch and drainage were performed. Associated actions as suture of the pulmonary parenchyma, binding of the internal thoracic arteries and the intercostal pedicle and suture of the diaphragmatic wounds were done.

We regret only one death during the operation with a patient having a 3 cm wound of the left ventricle. The average stay in intensive care unit was 4 days. Immediate post-operative complications were observed in 6 patients: 3 cases of SDRA, an ischemic case of colitis, a case of thrombosis of superior vena cava, a case of cerebral anoxia with muteness. The average duration of hospitalization was 15 day and all patients were seen regularly after hospital discharge with a median follow-up of 6 years. 5 patients had a functional symptomatology with atypical thoracic pains among 3 patients and effort dyspnea stage 2 among 2 patients. 5 patients kept electric after-effects: complete right block of branch among three patients, and incomplete in two patients. 14 patients had a cardiac echography which was normal among all these patients.

## Discussion

In this present series of penetrating cardiac injuries, all patients had stab wounds. In agreement with other series, our results have shown that victims of penetrating cardiac injuries are predominantly young males as it is found in urban violence. However, hospital mortality in our series was lower than what is usually reported, but still higher than in the cases of penetrating chest wounds without cardiac injuries.

The confirmation of the diagnosis of penetrating cardiac injury may be difficult especially in patients with unstable hemodynamic status. It must be kept in mind that all patients presenting with a precardial wound should be suspected of having a cardiac laceration [1]. This does not exclude that the wound can lie outside the confines of the “box”.

“Scoop and go” is an absolute prehospital principle of the management of patients suspected to have penetrating cardiac injuries. Only 4% of patients arresting in the ambulance survived, but 74% of the patients who arrived to the operating room and underwent thoracotomy survived. Clearly, these injuries are ominous and require rapid transfer [2].

Patients with penetrating cardiac injuries can be classified into five groups: lifeless, critically unstable, cardiac tamponnade, thoraco-abdominal injury and those with a benign presentation [3]. 42% of our patients belonged to the second group, 21% to the third group and 37% to fifth group.

As with all trauma patients a rapid, yet thorough, physical examination is mandatory. Location of wounds, assessment of cardiorespiratory status, heart and lung sounds and, in haemodynamically stable patients, a portable chest radiograph can be expeditiously performed. Penetrating cardiac trauma may result in exsanguinating hemorrhage or cardiac tamponnade. The rapid accumulation of even a small amount of blood causes increased intrapericardial pressure resulting in decreased venous return, decreased cardiac output, hypotension and, ultimately death. Beck's triad of muffled heart sounds, hypotension and jugular venous distension is the classic description of the signs of cardiac tamponnade but is not frequently present. The data on the survival benefit from tamponnade remains discordant. While some studies demonstrated increased survival with tamponnade [4], other reports have shown no benefit [5,6]. On the other hand, it is admitted that the cardiovascular condition of the victims and their consciousness status upon arrival to the hospital are significant predictors of outcome in penetrating cardiac injuries. In case of hemodynamic instability, no preoperative investigations are mandatory. Chest radiographs are of limited value in the initial assessment of the patient with penetrating cardiac injury.

The cardiac silhouette is not enlarged in about 80% of patients in acute cardiac tamponnade. At least 250 ml of pericardial fluid must be present to detect heart enlargement radiographically. It was detected only for 23% of cases in our series. The electrocardiogram may suggest tamponnade if QRS voltage is decreased or if the dominant QRS axis constantly changes (electrical alternans) secondary to the heart “floating” in the pericardium. ST segment elevation may be observed. A normal electrocardiogram (ECG) does not rule out a cardiac injury [2].

Historically, sub-xiphoid window was the gold standard to look for haemopericardium but, there are reports stressing on the risk of subxiphoid exploration like catastrophic hemorrhage or waste of valuable resuscitative time [7]. Echocardiography has now become the modality of choice [8]. In a prospective, multicentric study of ultrasound in patients with possible cardiac wounds, Rozycki and colleagues reported 100% sensitivity, 97% specificity, 97% accuracy [9] and mean time from study to operation of 12 minutes [5]. Echocardiography was done to 6 of our patients and was positive in 5 cases. In stable patients the sensitivity, specificity and accuracy of computed tomography (CT) in detecting hemopericardium is highly reliable. However, management of patients during CT examination may be very difficult if these patients deteriorate; then echocardiographic examination with a portable device is the preferred method [7].

**Figure 3:**
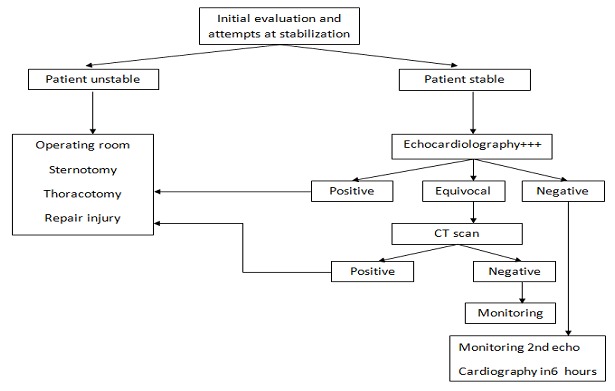
Algorithm of initial assessment of suspected cardiac injury

An algorithm for the initial assessment of penetrating injuries is presented (Figure 3). Patients with penetrating cardiac injuries can be presented very quickly to the operating room and can be reasonably stable or absolutely moribund, so it is difficult to make blanket recommendations. The value of fluid resuscitation should never be underestimated. Filling pressures of 25 to 30 mmHg may be necessary to oppose the resistance of heart filling presented by an expanding pericardial volume. General anesthetic induction is potentially catastrophic until the bloody pericardial effusion is released. Paralysis and endotracheal intubation should be undertaken thoughtfully, as positive pressure ventilation reduces right ventricular preload and can decrease cardiac output acutely [2].

The choice of incision may be influenced by the surgeon's experience. While a posterolateral thoracotomy affords excellent exposure of the pleural cavity it allows only limited cardiac exposure. Another disadvantage is it may exacerbate haemodynamic instability, since the lateral decubitus position can result in worsening hypotension [5]. Median sternotomy affords optimal mediastinal exposure, it can be performed rapidly and allows repair of the cardiac injury. While cardiothoracic surgeons have more experience with this approach, a well trained general surgeon is more than capable of using it to treat cardiac injuries. Left antero-lateral thoracotomy was our approach of choice for emergency room thoracotomy.

The anatomic position of the heart within the thorax determines to some degree the injury encountered. The right and left ventricles each, are injured about 40% of the time. The frequency of right atrial injury is 24% and that of left atrial injury 3%. For our series, 74% of patients had right ventricular injury, 10% had left ventricular injury and 5% of them had right atrial injury.

Once the mediastinum has been exposed the pericardium must be opened. Several techniques are available to achieve temporary control of the cardiac injury including digital control, placement of a Foley catheter and the use of skin staples. We favour digital control since it is a simple and direct method. It does not need completely stop cardiac bleeding but rather decrease it and facilitate definitive repair [5]. Cardiac bleeding is best controlled by using 3/0 prolene on a large, round-bodied needle to go through both sides of the laceration in one pass of the needle. The two ends of the suture are pulled upwards with one hand, greatly reducing bleeding as the edges of the wound are approximated; the needle is then re-inserted to complete a figure-of-eight stitch.

The pericardium can be left open after placing mediastinal and thoracic drains. Closure of anterolateral thoracotomy is easy; the sternal part is easily approximated with one wire, while the thoracic portion of the incision is closed in the standard fashion.

Occasionally, lesions are recognized days after the initial injury. These are often intracardiac shunts, valvular decompensation (manifesting as hypotension, pulmonary edema, and new murmurs), and ventricular aneurysms. Echocardiography and cardiac catheterization are often needed to characterize the abnormalities. The patient may also develop the postpericardiotomy syndrome, manifesting as fever, chest pain, pleural effusions, pleural rub, and an ECG consistent with pericarditis (diffuse ST segment abnormalities). These patients are often treated with non-steriodal anti-inflammatory drugs [2].

## Conclusion

Penetrating cardiac injuries are one of the leading causes of death from urban violence. Outcome after penetrating cardiac injuries varies widely depending on the injury's mechanism, anatomic injury location, and physiologic status. The management of these injuries is possible in a general surgery department, but it requires a rapid prehospital transfer, a yet thorough physical examination along with efficient surgical management, all done in minimal time.
